# Prediction of long‐term treatment outcome in Alzheimer's disease based on EEG connectivity

**DOI:** 10.1002/alz.085157

**Published:** 2025-01-09

**Authors:** Tak Youn, Hea Jeong Park, Hyong Sook Jun

**Affiliations:** ^1^ Dongguk University International Hospital, Goyang, Gyeonggi Korea, Republic of (South); ^2^ Yonsei University College of Medicine, Department of Nuclear Medicine, Seoul, Seoul Korea, Republic of (South); ^3^ Baehwa University, Seoul, Seoul Korea, Republic of (South)

## Abstract

**Background:**

It is difficult to predict of long‐term treatment response of AD to medical treatment when starting medication. We explored EEG brain connectivity as a potential biomarker for long‐term medication outcomes in patients with AD.

**Methods:**

Resting‐state EEG was recorded from a total of 56 AD patients (mean age = 73.23±6.39) when starting medication after diagnosis of AD. EEG was recorded in 32 channels. We divided the patients into two groups, good and poor respondent groups according to clinical evaluation after treatment and dosage changes of AD medication with MMSE score change. Starting dosage of AchE inhibitor, donepezil was 7.75±3.85mg and the mean treatment period is 104.05±57.93 weeks. The mean final medication dosages are donepezil 13.23±6.93mg and memantine 9.52±9.68mg. Initial MMSE scores of good respondent group (n=32, M=11, F=21) is 20.06±4.35 and poor respondent group (n=24, M=7, F=17) is 19.63±4.07. We analyzed brain EEG connectivity among the 246 cortical regions defined by the Brainnetome atlas.

**Results:**

Our study found that the difference in brain EEG connectivity, especially intrahemispheric regional connectivity in gamma frequency bands, is increased at multiple regions of right hemisphere such as between Brodmann Area 44 and 39, such as DLFPC and hippocampal areas, DLFPC and caudate nucleus, middle frontal and inferior parietal areas in poor respondent group (p<0.00001). In alpha frequency band, EEG connectivity between right DLPFC(BA 9/46) and right paracentral lobule of frontal lobe (BA 4) is increased in good respondent group (p<0.0001). In theta frequency band, EEG connectivity in the areas of frontal lobe (BA 4 and 6) is decreased in good respondent group (p<0.0001).

**Conclusions:**

This study shows increased gamma bands EEG connectivity between right anterior and posterior areas in poor respondent group. In general cortical gamma‐band activity is increased by selective attention in normal populations and related to cholinergic system. Elevated gamma band power was observed in AD when compared to MCI and control subjects was reported in a previous study. Our results suggest the potential of the gamma EEG connectivity of initial EEG when starting treatment as a biomarker to predict long‐term medical treatment for AD medication.

